# Local interactions and global properties of wild, free-ranging stickleback shoals

**DOI:** 10.1098/rsos.170043

**Published:** 2017-07-12

**Authors:** Ashley J. W. Ward, Timothy M. Schaerf, James E. Herbert-Read, Lesley Morrell, David J. T. Sumpter, Mike M. Webster

**Affiliations:** 1 School of Life and Environmental Sciences, University of Sydney, Sydney, Australia; 2 School of Science and Technology, University of New England, Armidale, Australia; 3 Department of Mathematics, Uppsala University, Uppsala, Sweden; 4 Department of Biology, Stockholm University, Stockholm, Sweden; 5 School of Biological, Biomedical and Environmental Sciences, University of Hull, Hull, UK; 6 School of Biology, University of St Andrews, St Andrews, UK

**Keywords:** collective behaviour, schooling, grouping

## Abstract

Collective motion describes the global properties of moving groups of animals and the self-organized, coordinated patterns of individual behaviour that produce them. We examined the group-level patterns and local interactions between individuals in wild, free-ranging shoals of three-spine sticklebacks, *Gasterosteus aculeatus*. Our data reveal that the highest frequencies of near-neighbour encounters occur at between one and two body lengths from a focal fish, with the peak frequency alongside a focal individual. Fish also show the highest alignment with these laterally placed individuals, and generally with animals in front of themselves. Furthermore, fish are more closely matched in size, speed and orientation to their near neighbours than to more distant neighbours, indicating local organization within groups. Among the group-level properties reported here, we find that polarization is strongly influenced by group speed, but also the variation in speed among individuals and the nearest neighbour distances of group members. While we find no relationship between group order and group size, we do find that larger groups tend to have lower nearest neighbour distances, which in turn may be important in maintaining group order.

## 1. Introduction

The collective movements of animal groups provide some of the most striking examples of self-organization in nature. Individuals in flocks, schools, herds and swarms show co-ordination without choreography. Collective motion can be broadly studied at two interdependent levels, the individual and the group. Group-level organization may be measured in terms of the morphology of the group, the patterns of distribution of animals within the group and the overall co-ordination of group members. The shape of an animal group emerges through self-organization, mediated by environmental factors. For example, the speed at which the group travels is known to affect its overall morphology [1,2]. Interestingly, while similar environmental pressures may cause convergence of group morphology across species, there may be enough difference in group morphology between species to allow them to be differentiated on this basis [3,4]. Related to this, the distribution of animals within groups has implications for the functioning of the group and for the benefit that individuals obtain from group membership [5,6]. Evidence suggests that animals within groups adopt positions that optimize effective communication with near neighbours [7,8]. The extent to which the group exhibits co-ordination, or order, is often measured as polarization. Highly polarized groups may function to increase the perceptual confusion experienced by a predator encountering such groups [9], and may additionally facilitate the spread of information between individuals [6,10,11]. Theoretical and empirical studies of collective motion have emphasized factors such as increase in speed and density in generating group polarization [12–15]. Despite breakthroughs in our understanding of these aspects of global organization in animal groups, it remains a priority to give broad consideration to the many potential factors that may be involved in this process in free-ranging conditions.

Global patterns observed in animal groups during collective motion are self-organized phenomena arising from the local interactions that occur between group members. Characterizing the distribution of animals within groups and the interactions that occur between them has been the focus of considerable research effort, with the goal of understanding how these interactions scale to produce global patterns [16–23]. Most of the empirical investigations of collective motion in fish shoals have been carried out in the laboratory, often under quite artificial conditions. While laboratory studies have yielded major breakthroughs in the study of collective motion, developing a robust understanding of the topic requires data on free-ranging animals in their natural environment. Of the studies that have been carried out on free-ranging animal groups, few have been able to examine the collective motion of many independent groups. Here, we examine data on naturally occurring shoals of stickleback to resolve questions regarding both group-level and individual-level patterns. First, we examined group-level patterns, determining the effects of group size, nearest neighbour distance and speed on polarization, aspect ratio and spacing behaviour. We also examined patterns within groups, in particular how the position of animals from the front to the back of the group, and from the centre to the edge, related to factors such as speed, size and near-neighbour densities. Finally, we examined the distribution of animals in free-ranging shoals relative to one another, and the influence that group members exert on one another, particularly in respect of matching speed, orientations and size, and the distance over which this influence may be measured.

## 2. Material and methods

We filmed shoals of sticklebacks in the Great Eau, a river near Saltfleet in Lincolnshire, UK (53°22′11.34′ N, 0°11′22.16′ E), over five consecutive days in August 2012, between the hours of 10.00 and 15.00 h. At the time of filming, the width of the channel was approximately 10 m. At the precise location of filming, the river has been canalized and the banks are formed from concrete. The depth in the section of channel that we filmed was 1.65 m, the flow speed at the surface was approximately 1.5–2.5 cm s^−1^ and the water temperature during filming ranged between 17.5 and 18.5°C. There was no submerged vegetation at the top of the water column where we filmed shoals of stickleback. At the time, underwater visibility was excellent, which facilitated the use of cameras for filming. The river supports populations of piscivorous fish, including pike (*Esox lucius*) and perch (*Perca fluviatilis*), as well as other fish species, including chub (*Squalius cephalus*), roach (*Rutilus rutilus*) and three-spine stickleback (*Gasterosteus aculeatus*), the focus of this study. At the time of filming, there were large numbers of young-of-the-year sticklebacks forming single species shoals at the water surface.

**Figure 1.**
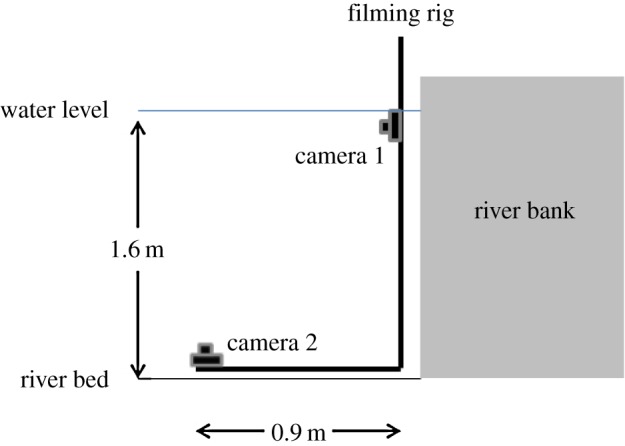
Schematic of filming apparatus used.

Filming was carried out by attaching two Panasonic FT-4 cameras to an L-shaped aluminium rig (figure 1). One camera was positioned to film along a horizontal plane, facing away from the river bank. A second camera was positioned on the river bed, at 90° to the vertical concrete river bank, filming upwards through the water column. The cameras were calibrated, both temporally and spatially, by passing an object of known size through the field of view. We filmed in 30 min sections at a resolution of 1080p and a frame rate of 25 frames per second. Following filming, we examined the videos to find instances where shoals crossed the field of view of the camera that was placed on the river bed. We then cross-referenced that film using the film taken from the camera filming at the water surface. From this, it was determined that the shoals in every case swam in a narrow, approximately two-dimensional plane at the water surface. As the maximum depth, from top to bottom, of these shoals was less than 50 mm (less than the water depth of many studies of collective behaviour in fish in laboratories), we used only the film taken from the camera on the river bed for the subsequent analysis. Finally, to minimize the risk of pseudo-replication, we spaced our sampling so that at least 15 min elapsed between sampling events.

We processed the film using the VirtualDub editing software, cutting the footage into sections featuring shoals. We defined a shoal as a group of two or more fish where each fish was within 100 mm (approx. four body lengths) of at least one other fish. Furthermore, all shoals had to be more than 100 mm from the river bank, and more than 100 mm from the margins of the camera's field of view to ensure that we were filming discrete shoals and not sections of shoals. We converted those sections into the ‘.avi' format and tracked the shoals using the CTrax automated tracking software, subsequently correcting any errors using the FixErrors GUI [24]. All video analysis was performed blind by a research assistant who was not aware of the parameters of interest. Following this, we were then able to calculate individual-level properties, such as body length, speed, orientation, nearest neighbour distance and shoal position, and group-level properties, such as polarization, shoal structure and aspect ratio. Polarization and speed were determined using short time-series data, where we analysed 1 s of shoal movement, i.e. 25 frames, from the time that the maximum number of group members were simultaneously visible on screen. Shoal structure was calculated as the average ratio between the distances to the second-nearest neighbour and the nearest neighbour [11]. In a uniform lattice, this ratio is 1, and hence the closer the value is to 1, the more evenly spaced the animals are within a group. Aspect ratio is a simple metric to describe group morphology and is calculated as the ratio between the length of the shoal, which we determined as the extent of the shoal parallel to the mean facing direction of shoal members, and its width, which we measured as the extent of the group perpendicular to the mean facing direction. Further details on our methods for estimating body length, individual velocity (and hence speed), neighbour distances, within-shoal positions, polarization and group aspect ratio are provided in the supplementary information.

### 2.1. Data analysis

To examine differences among groups in terms of their global properties, we examined shoals containing four or more fish that were travelling upstream (see the electronic supplementary material for details of how we estimated the current's velocity). By selecting only those travelling upstream (defined as 180° ± 45° to the direction of the current), we were comparing groups that experienced conditions as similar as possible to one another. A total of 53 separate shoals fulfilled these criteria, ranging in number from 4 to 44 (see electronic supplementary material, table S1). We first examined the effect of the current on group polarization. To do this, we calculated the difference in degrees between the mean group travelling direction and the direction of the current, so that a difference of 180° means that the shoal is travelling directly against the current. We then used this as the independent variable in a regression analysis, and used polarization as the dependent variable.

Next, we examined which factors predicted the global properties of the group. The predictor variables were group size, mean group speed, the variance of the speed (calculated as the coefficient of variance of the speed, because absolute variance increases with speed) and mean nearest neighbour distance. The outcome, or dependent, variables were three global group properties, or patterns: polarization, aspect ratio and shoal structure. Many of the variables were not normally distributed, hence we transformed them prior to analysis. We examined each of these dependent variables in turn using multiple regression. We used collinearity diagnostics to ensure that the results from each individual predictor were robust and a Durbin–Watson procedure to test the assumption of independent errors. We also plotted the residuals to examine issues including heteroscedasticity. The data met all assumptions tested.

To examine patterns of local organization within shoals, we used all shoals comprising more than 10 fish, a total of 33 shoals (table 1). First, we determined the relative frequency that each fish encountered other individuals within the group at given relative coordinates. In addition, we determined the mean speed, mean relative alignment and mean absolute difference in speed (between focal fish and their neighbours) of fish as a function of the relative locations of their partners. These data are rendered as heat maps in two dimensions (see the electronic supplementary material for more details).

**Table 1.** Linear mixed regression models examining the effect of topological neighbour distance from the nearest neighbour to the tenth nearest neighbour on absolute differences between the body lengths, speeds and orientations of focal fish and neighbours. *p*-Values are corrected for multiple comparisons according to the Benjamini–Hochberg method. *N* = 33 groups.

		value	s.e.	*N*	*T*	adj. *p*
topological neighbour distance	body length	0.044	0.017	330	2.697	0.007
	speed	0.429	0.07	330	6.179	<0.001
	orientation	0.42	0.077	330	5.45	<0.001

In addition, we examined the relationship between the size, speed and orientation of a focal fish and its neighbours as a function of topology. To do this, we calculated the difference between a focal fish and its near neighbours and plotted this as a function of distance, from the nearest neighbour, second nearest neighbour and so on up to the tenth nearest, using regression analysis. As many of the variables were not normally distributed, we performed log transformations. We tested this using linear regression analysis, incorporating a Durbin–Watson procedure to test for the independence of the errors. To account for the use of multiple tests, *p*-values are adjusted following Benjamini & Hochberg's [25] method for false discovery rate control.

Next, we investigated whether there were relationships between an animal's relative position in the group and its size and behaviour. We scaled each individual's position in the group from front to back between 0 and 1, so that the individual at the front of the group was given a score of 0 and the individual at the back was given a score of 1. We performed a similar process for the positions from the centre (nearest to the centroid) to the edge (farthest to the centroid), again scaling the values so that, in each case, they ranged from 0 at the centroid to a score of 1 for the individual furthest from the centroid. We used these values as predictors of size, speed, nearest neighbour distance, local density and angular deviation. We defined local density as the number of near neighbours within three body lengths of a focal individual. This criterion was adopted on the basis of our previous studies on this population of sticklebacks [26,27]. Moreover, the results remained consistent regardless of whether we used larger (four body lengths) or smaller (two body lengths) criteria for local density. Angular deviation is the absolute difference between the direction of motion of a focal fish and the mean travelling direction of the shoal. These data were analysed using linear mixed-effect models in R including shoal as a random factor. Normality was assessed through visual inspection of quantile–quantile plots and plots of standardized residuals against fitted values. Again, we applied the Benjamini–Hochberg procedure to control for the false discovery rate.

## 3. Results

In total, we examined 53 shoals, comprising 868 fish. The body length of the fish in the study was 22.3 ± 4.7 mm (mean ± s.d.).

### 3.1. Global patterns

The shoals in the study were highly polarized. Although we initially assumed that this arose primarily through the fish responding to the current in the river, rather than through local interactions within the groups, our data indicate that the group direction of travel relative to the current was only weakly related to group polarization (linear regression: *r*^2^ = 0.05, *F*_1,51_ = 2.688, *p* = 0.11).

**Figure 2.**
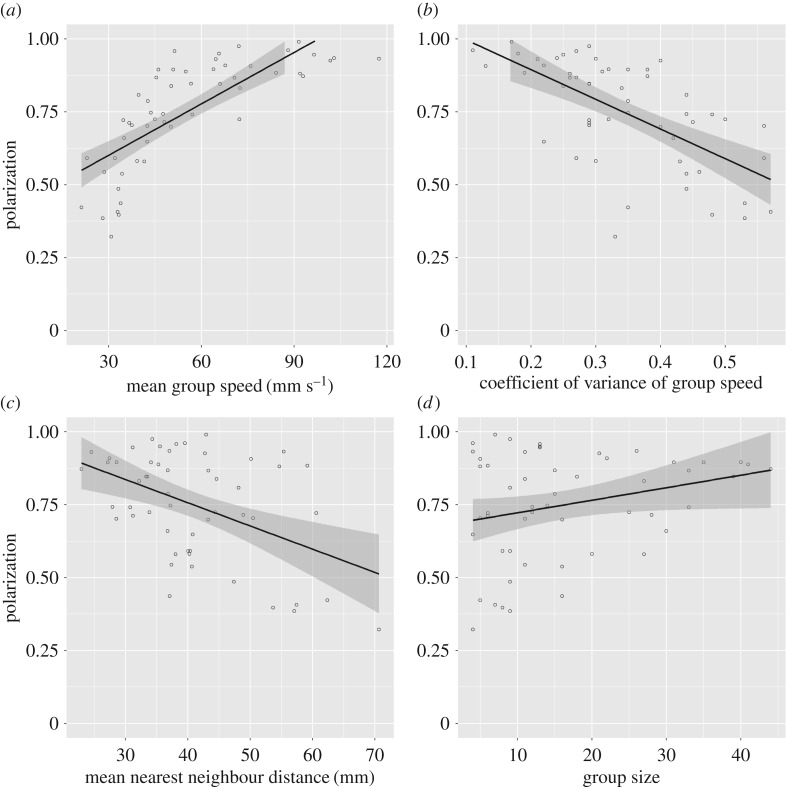
Plots showing the relationship between group polarization and (*a*) mean group speed, (*b*) the coefficient of variance of group speed, (*c*) mean nearest neighbour distance and (*d*) group size. *N* = 53 groups.

Group polarization could be predicted on the basis of the multiple regression model (*r*^2^ = 0.816, *F*_4,48_ = 53.3, *p* < 0.001). Of the predictor variables, mean group speed (standardized B = 0.604, *t* = 7.77, *p* < 0.001), the coefficient of variance of speed (standardized B = −0.338, *t* = −4.329, *p* < 0.001) and mean nearest neighbour distance (standardized B = −0.201, *t* = −2.491, *p* = 0.016) explain a significant amount of the variation, but not group size (standardized B = 0.026, *t* = 0.309, *p* = 0.759). Groups that move more quickly, with relatively lower variation among members' speeds and with relatively low near-neighbour distances tend to be more polarized (figure 2).

**Figure 3.**
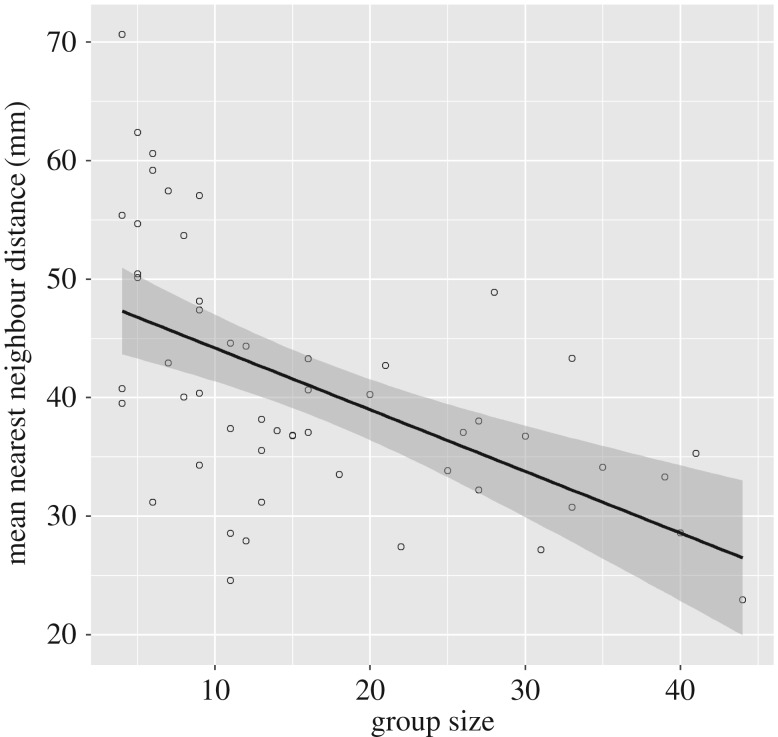
Plot showing the relationship between group size and mean nearest neighbour distance. *N* = 53 groups.

The mean nearest neighbour distance was predicted by a regression model (*r*^2^ = 0.416, *F*_3,49_ = 11.643, *p* < 0.001). Group size (standardized B = −0.641, *t* = −5.236, *p* < 0.001) but not mean group speed (standardized B = −0.246, *t* = −1.811, *p* = 0.076) or the coefficient of variance of group speed (standardized B = 0.154, *t* = 1.032, *p* = 0.307) explained a significant amount of the variation. The mean nearest neighbour distance decreases with increase in group size, but is unaffected by the mean or standard deviation of group speed (figure 3).

Group structure was also predicted on the basis of the multiple regression model (*r*^2^ = 0.211, *F*_3,45_ = 4.016, *p* = 0.013). The coefficient of variance of speed (standardized B = −0.381, *t* = −2.308, *p* = 0.026), but not mean group speed (standardized B = 0.12, *t* = 0.7, *p* = 0.487) or group size (standardized B = −0.039, *t* = −0.271, *p* = 0.787), explained a significant amount of the variation. Groups with lower variation in speed were more structured, which is to say they were spaced more evenly.

Finally, the aspect ratio of the group could also be predicted on the basis of the multiple regression model (*r*^2^ = 0.322, *F*_4,48_ = 5.689, *p* = 0.001). Of the predictor variables, mean group speed (standardized B = 0.564, *t* = 3.775, *p* < 0.001), but none of the others (coefficient of variance of the group speed (standardized B = 0.137, *t* = 0.914, *p* = 0.366), group size (standardized B = −0.021, *t* = −0.129, *p* = 0.898) or nearest neighbour distance (standardized B = −0.169, *t* = −1.092, *p* = 0.28)) explained a significant amount of the variation. Groups that move more quickly tend to have a greater aspect ratio, which is to say that they are relatively more elongated.

### 3.2. Local organization

**Figure 4.**
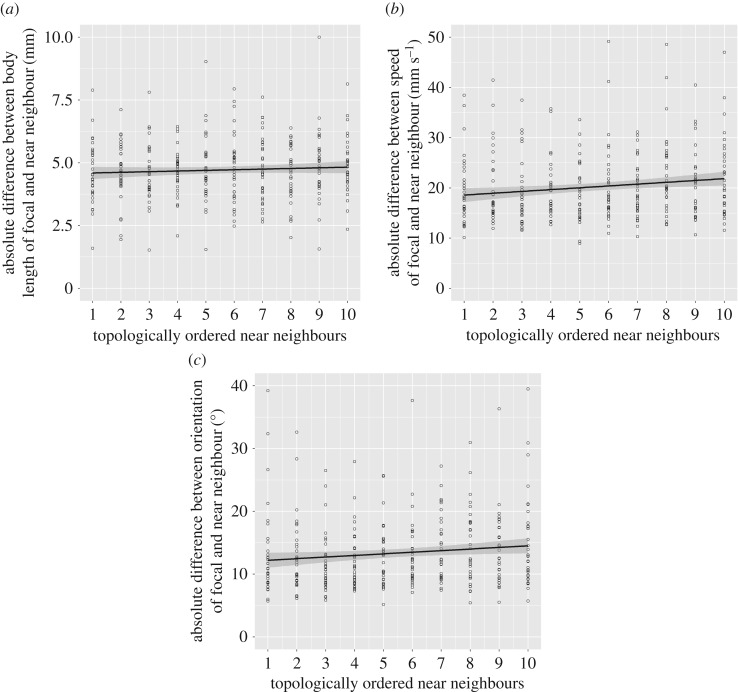
Plots showing the absolute differences between focal fish and their neighbours in (*a*) body length, (*b*) speed and (*c*) orientation as a function of topological distance from the focal fish, from its nearest neighbour to its tenth nearest neighbour. *N* = 33 groups.

The mean absolute difference between focal fish and other shoal members in terms of size, speed and orientation increased with increase in topological distance from the focal fish (table 1 and figure 4).

**Figure 5.**
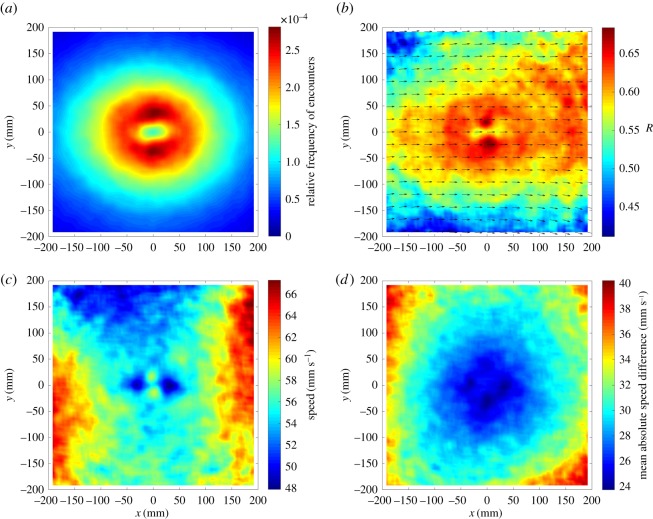
Heat plots showing (*a*) the distribution of neighbours, (*b*) the mean relative direction of motion of neighbours (arrows) and polarization between focal fish and neighbour velocities, *R*, (*c*) the speed of focal fish as a function of relative neighbour positions (mean speed across all fish = 58 mm s^−1^) and (*d*) the mean absolute difference in speed between neighbouring fish and focal fish, all with respect to a focal fish centred at the origin and travelling parallel to the positive *x*-axis in groups of 10 or more sticklebacks, *N* = 33 groups, 740 individual fish.

We plotted a distribution density plot across all groups of greater than 10 members with respect to a focal fish centred at the origin and travelling along the *x*-axis (figure 5*a*). In addition, we plotted alignment (figure 5*b*), speed (figure 5*c*) and absolute difference in speed between neighbouring fish and focal fish (figure 5*d*) as a function of the position of neighbours relative to a focal fish.

**Figure 6.**
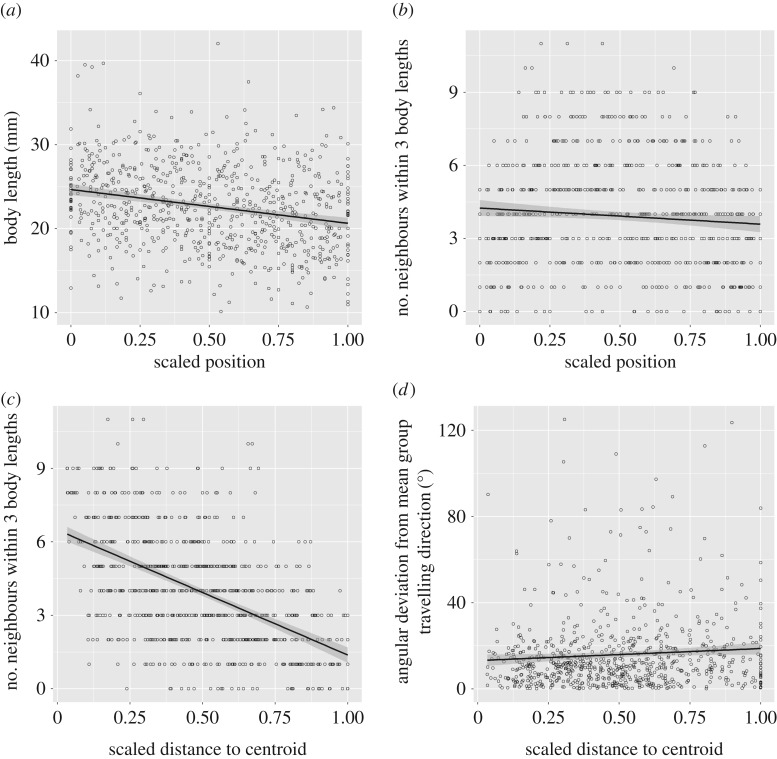
Plots showing relationships between (*a*) scaled position in the shoal from front (scored as 0) to back (scored as 1) and body length; (*b*) scaled position and local density, defined as the number of neighbours within three body lengths of a focal fish; (*c*) scaled distance to the centroid (where 0 is the centroid and 1 represents the furthest fish from the centroid) and local density; and (*d*) scaled distance to the centroid and angular deviation of the travelling direction of a focal fish from the group's mean travelling direction. *N* = 33 groups, 740 individual fish.

Larger individuals tend to occupy positions towards the front of groups, although we found no evidence to suggest that these individuals are faster swimmers based on our short time series. Local density was greatest towards the front of the shoal and decreased towards the back of the shoal (table 2 and figure 6).

**Table 2.** Linear mixed regression models examining, first, the relationship between standardized position from the front to the back of the group and speed, nearest neighbour distance, local density and angular deviation, and, second, the relationship between standardized distance to centroid from the centre to the periphery of the group and the same variables are corrected for multiple comparisons according to the Benjamini–Hochberg method.

	value	s.e.	*N*	*T*	adj. *p*
position					
size	−4.019	0.523	740	−7.68	0.001
speed	−3.27	2.598	740	−1.259	0.297
NND	1.144	2.069	740	0.696	0.607
local density	−0.661	0.255	740	−2.591	0.019
angular deviation	0.651	2.032	740	0.321	0.748
distance to centroid					
size	0.24	0.682	740	0.35	0.83
speed	−7.206	3.265	740	−2.207	0.046
NND	26.137	2.376	740	11	0.001
local density	−5.164	0.257	740	−20.07	0.001
angular deviation	6.789	2.514	740	2.7	0.018

Local density was greatest in the centre of groups and decreased with increase in distance from the centroid. Correspondingly, fish positioned towards the edges of groups had significantly larger nearest neighbour distances. Angular deviation increased with increase in distance from the centroid, while there was a marginal tendency for fish that were further from the centroid to swim more slowly (table 2 and figure 6).

## 4. Discussion

The free-ranging shoals of sticklebacks showed characteristic patterns of near-neighbour distribution with high encounter frequencies at a distance of approximately one to four body lengths from a central focal fish, with distinct peaks alongside the fish. Collision avoidance in theoretical models of collective behaviour is often achieved by short-range repulsion [17]. The existence of such a rule may be inferred from the clear, low encounter-frequency area of within one body length of the focal fish [28]. At distances beyond four body lengths, the frequency of neighbour interactions is decreased, although neighbours beyond these distances, and particularly those in front of a focal fish exert considerable influence on the speed and orientation of that fish, which tends to suggest that the flow of information in these moving groups occurs from the front of the group to the back [29,30]. The lateral positioning behaviour is similar to that reported in other fish species in laboratory experiments [11,31]. Interestingly, these lateral encounter-frequency peaks coincide with the areas in which the fish appear to align most closely with neighbours, and where the mean absolute differences in speed are least. In addition, the topological analysis revealed local organization in the shoals where near neighbours tend to be more closely matched in size, speed and orientation than more distant neighbours. Furthermore, the analysis revealed clear areas where near neighbours were positioned behind and, particularly, in front of the focal fish, where the speed of the focal fish is decreased. This seems to highlight the importance of speed, as well as orientation, in regulating the interactions in groups [32,33]. Broadly, there is considerable congruence between the basic interaction patterns revealed here in free-ranging fish shoals and in shoals of fish studied in comparable ways under laboratory conditions (although here we examine speed, rather than some form of change in speed, as examined elsewhere [32,33]).

Collective motion is often characterized by a high degree of group-level order. Simulation models and previous experimental work have highlighted the relationships between increase in speed and density with an increase in group polarization [12,14,34]. Our results similarly demonstrate the importance of speed to group order, but also point to the involvement of other parameters, particularly low variance in speed across the group and low near-neighbour distances. In a mensurative study such as this, we obviously cannot infer causal relationships. Resolving which of these parameters is cause and which is effect in relation to polarization would represent a useful step for further research.

Given the predictions of previous work, it is perhaps surprising that group polarization was unrelated to group size. Our results contrast with previous studies suggesting a decrease in group-level polarization with increase in group size [35,36]. Although we examined group sizes ranging from 4 to 44 individuals in number, it may well be that any loss of group-level order may occur at group sizes larger than the maximum examined here. Nonetheless, the relationship between density and group-level order, based on the organizing effect of higher interaction frequencies in denser aggregations, is reasonably well established [37–41], and these findings were replicated in the present study, in the sense that groups with smaller nearest neighbour distances were more polarized. We might speculate that because nearest neighbour distances were smaller in larger groups, this provides a mechanism for the maintenance of group-level order in these larger groups.

Speed mediates not only polarization but also other key aspects of global structure in groups. Faster groups had greater aspect ratios, which is to say that they were more elongated. One explanation for this is that as individuals speed up, they need to leave more space in front of them to avoid collisions, producing a more elongated group morphology [2]. There is also greater variation in speed between group members in faster groups, which may contribute to a reduction in the regularity of their spacing. Overall, these faster groups which tend to be highly ordered according to the measure of group-level polarization appear to be less ordered in respect of the consistency of speed and spacing across the group. This may mean that the efficient communication afforded by increased polarization in faster groups is mitigated to some degree by the reduction in regularity of spacing. It would be fascinating to conduct experiments examining communication across groups in specific relation to these parameters.

Local density was relatively greater at the front and the centre of groups than at the back and the periphery, respectively. These patterns are consistent with previous studies on animal groups (e.g. [35,42,43] and with the predictions of SPP models [44]) (although see also [3,45]). Models suggest that high frontal density may be a self-organized phenomenon, emerging as a by-product of the interaction rules followed by group members [38,39]. Animals at the edges of groups have greater domains of danger and consequently are thought to be at greater risk of predation [5,46–49] (although see also [50]). It is perhaps surprising, then, that such individuals also have larger nearest neighbour distances, which is likely to compound this problem. However, a number of studies have reported positional benefits for edge versus centre individuals in foraging and it is known that foraging and hungry animals tend to increase their distance to conspecifics, which potentially explains our observations [51,52]. Interestingly, animals towards the periphery of groups had both a higher angular deviation and relatively lower swimming speeds than those towards the centre. Taken together, this suggests that, in many cases, peripheral fish may be in the process of splitting from the group.

Larger fish tended to occupy positions at the front of the group. A similar pattern has been observed in other fish species [53,54]. This may emerge through the greater swimming speeds of larger fish, although this was not obviously apparent from the short time-series data that we present here. A consequence of this is that because leading animals in moving groups dominate decision-making, larger fish probably have greater influence on the group trajectory. In addition, smaller individuals, which have a higher cost of transport per unit distance, are potentially able to benefit from the hydrodynamic advantages of drafting [55–59]. This distribution of animals may also contribute to the size sorting that we observed within shoals, where near neighbours are more similar in size than animals that are further apart. This size sorting within shoals is probably based on both the active preference of fish and passive sorting, based on similarity of swimming speeds [60–62].

One of the main aims of the present study was to examine the collective behaviour of groups of fish in the wild, and to compare patterns both within and between those groups. Generally, there is considerable concordance between the patterns of local organization described in our study and previous, laboratory-based studies. Drawing firm conclusions regarding group-level patterns is in some ways more difficult, because although the patterns are easily observed, providing mechanistic or functional explanations is notoriously difficult [7,45,63]. Nonetheless, we again note considerable agreement between our findings and the predictions of theoretical modelling studies, and our findings provide a valuable point of comparison for laboratory studies while providing some clearly testable hypotheses that we recommend for future work.

## Supplementary Material

ESM 1

## Data accessibility

The datasets supporting this article have been uploaded as part of the supplementary material; they are also available via Dryad (http://dx.doi.org/10.5061/dryad.4kd16) [64].
