# Supplementary material for: Local interactions and global properties of wild, free-ranging stickleback shoals
Source: R Soc Open Sci. 2017 Jul 12;4(7):170043. doi: 10.1098/rsos.170043 (PMC5541530; doi:10.1098/rsos.170043)
Supplement: ESM 1 [file rsos170043supp1.docx]

**Local interactions and global properties of free-ranging stickleback shoals – supplementary information**

A J W Ward, T M Schaerf, J E Herbert-Read, L Morrell, D J T Sumpter and M M Webster

*CTrax output*

The tracking data from CTrax that we used for subsequent analysis included time series of the positions of each fish $i$, $\left(x_{i}(t), y_{i}(t)\right)$, in millimetres, two parameters $a_{i}(t)$ and $b_{i}(t)$ that corresponded to a quarter of the length of the major and minor axes of an ellipse that was fitted over the image of each fish for each time step (in millimetres) and the facing direction of each fish $\theta_{i}(t)$ relative to the positive $x$-axis .

*Body length, speed relative to observation point, polarisation, neighbour distances and shoal structure*

We estimated the body length of each fish as the median across time of $4a_{i}(t)$ across the entire time series that each individual was tracked.

For each shoal, we identified the first frame for which the maximum number of fish were simultaneously visible within the same shot. We extracted data corresponding to the frame were the maximum number of fish were visible, along with the following 25 frames (1 second) of data. We then performed the following calculations for this short time series of data (or subset of this data where specified).

We estimated the components of each fish’s velocity at a given time $t$ in the $x$- and $y$-directions respectively using the standard forward difference approximations:

$u_{i}(t) = \frac{x_{i}\left(t + \Delta t\right) - x_{i}\left(t\right)}{\Delta t}, v_{i}(t) = \frac{y_{i}\left(t + \Delta t\right) - y_{i}\left(t\right)}{\Delta t}$ (1)

where $\Delta t = 1/25$seconds was the duration between consecutive video frames. We then estimated each visible group member’s mean speed as the mean of:

$$s_{i}(t) = \sqrt{\left(u_{i}(t)\right)^{2} + \left(v_{i}(t)\right)^{2}}$$

derived from the short sequence of 26 frames. To obtain a grand mean for each group, we took the mean of the individual swimming speeds of all group members.

We estimated the polarisation using the direction of motion of group members for the short time series. Polarisation in direction of motion (relative to the camera) was determined by first constructing unit vectors in the direction of motion of each fish, with components:

.

Polarisation in direction of motion at time $t$ was then calculated via:

,

which we then averaged over the 26 frames analysed for each shoal.

We determined the magnitude of the angle between the facing direction and the direction of motion of each fish, given by:

,

where $f_{i, x}(t) = \cos\left(\theta_{i}(t)\right)$ and $f_{i, y}(t) = \sin\left(\theta_{i}(t)\right)$ were the $x$- and $y$-components respectively of a unit vector pointing in the facing direction of fish $i$ at time $t$.

Subsequently, we determined the mean of $\phi_{i}(t)$ for each fish across all times $t$ for which we had estimated the fish’s velocity (25 consecutive frames in total). Using standard methods of circular statistics [1, 2], the mean of $\phi_{i}(t)$ was given by:

$\bar{\phi}_{i} = \text{atan2}\left(S, C\right)$,

where $C = \sum\cos\left(\phi_{i}(t)\right)$, $S = \sum\sin\left(\phi_{i}(t)\right)$ and $\text{atan2}\left(S, C\right)$ was the four quadrant inverse tangent of $C$ and $S$, as implemented by the intrinsic MATLAB function of the same name, such that $-180^{\circ} < \text{atan2}\left(S, C\right) \leq180^{\circ}$.

We determined the distances between all fish, $i$, and their partners, $j$, for the initial frame of data where the largest number of shoal members were tracked using the standard distance formula:

$d_{i, j}(t) = \sqrt{\left(x_{j}(t) - x_{i}(t)\right)^{2} + \left(y_{j}(t) - y_{i}(t)\right)^{2}}$. (2)

From these distances we then determined the distance from each individual to the first to last nearest neighbours. As noted in the main text, shoal structure was examined via the mean across all shoal members of the ratio of the second nearest neighbour distance to the nearest neighbour distance for each fish (a measure suggested in [3]).

*Individual positions within shoal, shoal length, width and aspect ratio*

We determined the front to back order of individuals within the group for the key frame where the maximum number of shoal members were first visible, relative to the mean facing direction of individuals during the given frame. In addition, we determined the extent of the group parallel and perpendicular to the mean facing direction (hereafter we refer to the extent of the group parallel to the mean facing direction of group members as the length, $l$, of the group, and the extent of the group perpendicular to the mean facing direction as the width, $w$, of the group). We then determined the length to width aspect ratio of the group.

To determine individual positions within the group, we first identified the group centre $\left(X,Y\right)$ given by the mean of the $x$- and $y$-coordinates of all individuals visible during the key frame. We then calculated the average facing direction of all group members relative to the positive $x$-axis, $\bar{\theta} = \text{atan2}\left(F_{y}, F_{x}\right)$, where $F_{x} = \sum_{i = 1}^{n} f_{i, x}(t)$ and $F_{y} = \sum_{i = 1}^{n} f_{i, y}(t)$ ($f_{i, x}(t)$ and $f_{i, y}(t)$ were the $x$- and $y$-components of unit vectors in the facing direction of each fish, as defined above). Next, we shifted the coordinates of all fish such that the group centre was at the origin via $x_{i, s}(t) = x_{i}(t) - X$ and $y_{i, s}(t) = y_{i}(t) - Y$. We rotated the coordinates of all group members about the group centre in such a way that the mean facing direction was parallel to, and pointed in the same direction as the positive $x$-axis via the standard transformations:

.

After the shifting and rotation of coordinates, the fish at the front of the group (relative to the mean facing direction of individuals) had the greatest value of $\tilde{x}_{i}(t)$ and the fish at the back of the group had the least value of $\tilde{x}_{i}(t)$; we thus identified the front to back positions of group members by sorting the values of $\tilde{x}_{i}(t)$ in descending order. We assigned a standardised position score to the fish in position $k$ out of $n$ via $s = (k - 1)/(n - 1)$. Further, we identified the maximum and minimum values of both $\tilde{x}_{i}(t)$ and $\tilde{y}_{i}(t)$, denoted $\tilde{x}_{\max}$, $\tilde{x}_{\min}$, $\tilde{y}_{\max}$ and $\tilde{y}_{\min}$. We determined the group length, which we defined as , and the group width, which we defined as . Finally, we determined the length to width aspect ratio of the group, given by $l/w$.

*Estimates of stream velocity*

In addition to tracking fish, we estimated the motion of the stream within observation periods based on visual tracking of flotsam via CTrax (treating the flotsam as a passive tracer of local fluid motion). Within each of 10 distinct periods associated with observations of passing stickleback shoals we tracked the motion of 3 separate pieces of flotsam, deducing the associated coordinates $\left(x_{f}(t), y_{f}(t)\right)$ of the flotsam. As with the coordinates of the fish, we then estimated the components of velocity (relative to the fixed reference frame of the camera) and the speed of each piece of flotsam at each discrete time $t$ via:

$$u_{f}(t) = \frac{x_{f}\left(t + \Delta t\right) - x_{f}\left(t\right)}{\Delta t}, v_{f}(t) = \frac{y_{f}\left(t + \Delta t\right) - y_{f}\left(t\right)}{\Delta t} \text{and} s_{f}(t) = \sqrt{\left(u_{f}(t)\right)^{2} + \left(v_{f}(t)\right)^{2}}.$$

We determined the mean speed of each piece of flotsam across the entire duration of tracking, $\bar{s}_{f}$. We also estimated the mean direction of motion of the flotsam; to do this we constructed unit vectors in the direction of motion of the flotsam for each time $t$ with components:

.

We then summed these unit vectors across all tracked times $t$; the resultant vector then pointed in the mean direction indicated by the unit vectors. Algebraically, the mean direction of motion of the flotsam was given by $\bar{\theta}_{f} = \text{atan2}\left(V, U\right)$ where and . We then determined the mean of the mean speeds of the 3 pieces of flotsam tracked during the same observation period, $\tilde{s}_{f}$, and the mean of the mean directions of motion of the 3 pieces of flotsam, $\tilde{\theta}_{f}$. Finally, we approximated the motion of the stream within each distinct period as being constant and uniform everywhere, with speed $\tilde{s}_{f}$ in the direction $\tilde{\theta}_{f}$.

*Mean angles between direction of motion and the stream*

We determined the magnitudes of the angle between the direction of motion of each fish and our approximation to the direction of motion of the stream, $\tilde{\theta}_{f}$. The magnitude of the angle between a fish’s direction of motion and the stream was determined via:

.

We determined the mean of $\beta_{i}(t)$for each fish over the short sequence of frames starting from the key frame where the maximum number of group members were first tracked.

*Distributions of relative neighbour positions, relative neighbour alignment and speed as a function of relative neighbour positions*

Recent studies have examined the distributions of relative neighbour positions [4, 5, 6, 7, 8, 9], alignment with neighbours [5, 8, 9] and mean speed of individuals as a function of relative neighbour positions [8, 9] in moving animal groups (often rendered as heat maps). We examined the same measures for shoals of sticklebacks from our dataset where there were strictly greater than 10 individuals tracked within a shoal, taking into account the velocity of individual fish relative to the stream (rather than the fixed location of the camera, as was the case for the calculations described in previous sections of this supplementary material). This analysis also made use of the full time-series of tracking data, rather than the shorter sequences of data examined in earlier calculations.

We first smoothed the $x$- and $y$-coordinates of each fish’s trajectory using a Savitzky-Golay filter with span 5 and degree 2, as implemented by MATLAB’s intrinsic *smooth* function. We then determined the components of each fish’s velocity relative to the camera in the $x$- and $y$-directions respectively using equations (1). Additionally, we determined the $x$- and $y$-components associated with the stream’s mean speed and direction of motion, given by:

.

The components of each fish’s velocity relative to the stream were:

,

with associated speed, . We then constructed unit vectors in the direction of motion of fish relative to the stream with components:

.

Next, we performed a series of calculations to determine the location of each partner fish $j$ relative to the location and direction of motion relative to the stream of focal fish $i$ (treating every shoal member as a focal fish in turn). We determined the distance between each pair of fish for all times $t$ using equation (2). We then sought to calculate the angle between the direction of motion of each fish, $i$, and the straight line segment from the location of fish $i$ to the location of fish $j$. To determine the relevant angle, we first constructed a unit vector in the direction of the straight line segment from fish $i$ to fish $j$, which had components:

.

The magnitude of the angle between the direction of motion of fish $i$ relative to the stream and the vector pointing from fish $i$ to fish $j$ was then:

.

We performed an additional calculation to determine if partner fish $j$ were to the left or right of focal fish $i$. Relative to the direction of motion of fish $i$, fish $j$ lay to the left of fish $i$ if the sign of the following equation was positive:

.

If $\lambda_{i, j}(t)$ was negative, then fish $j$ lay to the right of fish $i$. The term inside the sign function in the above equation is the vertical component of the cross-product of the unit vector pointing in the direction of motion of fish $i$ (relative to the stream) with the unit vector pointing from fish $i$ to fish $j$. We defined the signed angle between the direction of motion of fish $i$ and the direction of motion of fish $j$ as:

$$\psi_{i, j}(t) = \left\{\begin{matrix} \lambda_{i, j}(t)\varphi_{i, j}(t) & if \lambda_{i, j}(t) \neq0, \\ \varphi_{i, j}(t) & if \lambda_{i, j}(t) = 0. \end{matrix}\right.$$

We then converted the relative locations of all partner fish from the polar form $\left(d_{i, j}(t), \psi_{i, j}(t)\right)$ to Cartesian coordinates using:

$$\begin{matrix} x_{i, j, \text{relative}}(t) = d_{i, j}(t)\cos\left(\psi_{i, j}(t)\right), \\ y_{i, j, \text{relative}}(t) = d_{i, j}(t)\sin\left(\psi_{i, j}(t)\right), \end{matrix}$$

such that focal fish were then located at the origin of the relative coordinate system, with their direction of motion relative to the stream parallel to and pointing in the direction of the positive $x$-axis.

We divided the domain centred on focal fish into a set of overlapping square bins, indexed $\left(l, k\right)$, such that the left edges of the bins were located at $x_{l, \text{left}} = -200, -196, -192, \ldots, 184$ (mm), the right edges of the bins were located at $x_{l, \text{right}} = -184, -180, -176, \ldots, 200$ (mm), the bottom edges of the bins were located at $y_{k, \text{bottom}} = -200, -196, -192, \ldots, 184$ (mm) and the top edges of the bins were located at $y_{k, \text{top}} = -184, -180, -176, \ldots, 200$ (mm). These bins extended 16 mm in both the $x$- and $y$-directions, and were separated by 4 mm in both the $x$- and $y$-directions. The overlapping bins were a means of smoothing the data (analogous to the moving window associated with standard moving average calculations) for visualisation purposes (no inferential statistical tests were applied to the output of the set of calculations described in this section).

For each focal fish $i$ and all partner fish $j$, across all video frames and shoals with more than 10 members, we tallied the number of times partner fish $j$were encountered within each bin. We then summed the tallies across all bins, and divided the tallies within each bin by this sum as an estimate of the relative frequency that partner fish were encountered at particular relative coordinates. Additionally, we included fish $i$’s speed from time $t$ in bin $\left(l, k\right)$ if $x_{l, \text{left}} < x_{i, j, \text{relative}}(t) \leq x_{l, \text{right}}$ and $y_{k, \text{bottom}} < y_{i, j, \text{relative}}(t) \leq y_{k, \text{top}}$ (across all partner fish $j$ at time $t$). In a separate set of bins, we also allocated the angular difference in directions of motion of fish $i$ and their partners $j$ to bin $\left(l, k\right)$ according to the above inequalities. Once all data had been allocated to bins, we determined the mean of the speed values contained within each bin, and the mean of the angular differences in direction of motion contained within each bin (using standard circular statistics as per [1, 2]). In the case of angular differences, we also determined $R$, which was a measure of the focus of the angles contained within each bin about the mean (within each bin) and identical to the polarisation of sets of angles within each bin (calculated in the same way as described in the section “*Body length, speed relative to observation point, polarisation, neighbour distances and shoal structure*” above). We rendered the relative frequency of encounters, mean speed and polarisation, $R$, as separate heat maps with MATLAB’s intrinsic *surf* function. Finally, we superimposed the mean difference in directions of motion as arrows on the plot of $R$.

In follow-up calculations we produced a further heat map to examine the mean absolute difference in speed between focal fish and their neighbours in given relative positions. The methodology for producing this heat map was identical to that described above, except that we allocated the absolute value of the speed of the neighbour fish $j$ minus the speed of the focal fish $i$ to bins.

SI Table 1: Descriptive statistics of the 53 free-ranging shoals of sticklebacks used in the analysis. Values for body length, speed and nearest neighbour distance are given as means with standard deviations in parentheses.

| **Ref ID** | **Group size** | **Aspect ratio** | **Polar** | **Body length** | **Speed** | **Mean NN distance** |
| --- | --- | --- | --- | --- | --- | --- |
| 1 | 5 | 3.514 | 0.881 | 22.38 (6.56) | 91.853 (23.9) | 54.67 (13.4) |
| 2 | 13 | 2.369 | 0.946 | 18.31 (4.33) | 96.4 (24.55) | 31.17 (17.44) |
| 9 | 22 | 1.166 | 0.909 | 20.9 (5.15) | 67.71 (15.14) | 27.42 (13.03) |
| 10 | 11 | 1.969 | 0.931 | 17.36 (1.94) | 64.63 (13.76) | 24.57 (9.26) |
| 13 | 40 | 0.817 | 0.896 | 27.73 (5.1) | 63.95 (20.67) | 28.58 (14.51) |
| 14 | 4 | 0.348 | 0.961 | 19.67 (4.07) | 88.07 (9.9) | 39.52 (8.9) |
| 16 | 4 | 2.653 | 0.932 | 21.5 (1.23) | 117.39 (35.3) | 55.37 (30.92) |
| 18 | 7 | 0.704 | 0.990 | 23.78 (6.34) | 91.47 (15.26) | 42.93 (13.68) |
| 20 | 31 | 1.161 | 0.895 | 24.17 (3.9) | 50.92 (19.39) | 27.17 (12.44) |
| 21 | 13 | 1.906 | 0.950 | 22.55 (3.18) | 65.73 (11.81) | 35.54 (12.38) |
| 24 | 11 | 0.513 | 0.702 | 24.3 (2.94) | 42.58 (23.87) | 28.55 (14.01) |
| 28 | 15 | 1.027 | 0.868 | 20.13 (3.66) | 45.45 (12.28) | 36.79 (10.29) |
| 30 | 41 | 1.055 | 0.888 | 20.51 (4) | 54.95 (17.09) | 35.29 (15.28) |
| 33 | 15 | 1.357 | 0.787 | 20.06 (4.8) | 42.7 (15.11) | 36.82 (17.56) |
| 34 | 8 | 1.164 | 0.591 | 20.92 (5.65) | 32.05 (8.6) | 40.07 (13.27) |
| 36 | 7 | 0.560 | 0.407 | 18.63 (5.73) | 32.91 (18.6) | 57.42 (20.35) |
| 37 | 9 | 1.223 | 0.975 | 19.8 (5.09) | 72.1 (20.93) | 34.3 (20.02) |
| 38 | 9 | 0.974 | 0.808 | 18.53 (2.66) | 39.74 (17.46) | 48.15 (20.52) |
| 42 | 5 | 0.534 | 0.422 | 23.47 (4.38) | 21.22 (7.49) | 62.36 (27.67) |
| 43 | 8 | 0.497 | 0.397 | 22.4 (4.02) | 33.31 (16.14) | 53.67 (14.07) |
| 44 | 6 | 0.704 | 0.721 | 20.2 (4.4) | 34.78 (10.18) | 60.59 (20.33) |
| 45 | 30 | 0.856 | 0.660 | 21.81 (4.55) | 35.05 (14.65) | 36.77 (17.61) |
| 48 | 13 | 2.132 | 0.958 | 21.57 (6.3) | 51.35 (13.72) | 38.16 (20.48) |
| 49 | 4 | 0.083 | 0.322 | 24.33 (5.15) | 30.88 (10.18) | 70.63 (30.17) |
| 50 | 20 | 0.622 | 0.581 | 24.2 (3.27) | 39.27 (11.75) | 40.26 (16.41) |
| 51 | 9 | 1.657 | 0.591 | 21.37 (4.07) | 23.02 (12.86) | 40.37 (12.11) |
| 52 | 39 | 1.787 | 0.847 | 22.43 (4.2) | 56.69 (16.26) | 33.32 (17.95) |
| 54 | 6 | 0.772 | 0.712 | 18.88 (2.4) | 36.7 (10.48) | 31.18 (5.64) |
| 55 | 33 | 1.461 | 0.741 | 21.06 (3.81) | 57.07 (27.59) | 30.76 (17.84) |
| 56 | 9 | 0.277 | 0.486 | 20.83 (5.89) | 33.15 (14.73) | 47.4 (8.2) |
| 57 | 16 | 1.029 | 0.436 | 22.1 (4.47) | 33.88 (17.93) | 37.08 (19.03) |
| 58 | 9 | 0.420 | 0.385 | 21.46 (5.73) | 28.11 (14.94) | 57.06 (23.88) |
| 59 | 6 | 0.539 | 0.884 | 25.82 (3.05) | 84.16 (15.99) | 59.17 (22) |
| 60 | 11 | 1.437 | 0.544 | 18.04 (4.77) | 28.66 (13.2) | 37.39 (17.66) |
| 61 | 5 | 0.890 | 0.704 | 22.76 (1.47) | 37.52 (10.85) | 50.45 (10.4) |
| 62 | 35 | 0.555 | 0.895 | 23.67 (5.06) | 46.1 (16.01) | 34.12 (13.72) |
| 63 | 28 | 0.933 | 0.715 | 22.55 (4.13) | 47.99 (21.53) | 48.89 (18.75) |
| 64 | 16 | 1.054 | 0.538 | 20.61 (5.4) | 34.35 (15.23) | 40.64 (19.74) |
| 65 | 16 | 1.598 | 0.699 | 20.32 (5.3) | 50.12 (19.8) | 43.27 (28.94) |
| 66 | 27 | 0.594 | 0.580 | 21.31 (5.31) | 41.54 (17.74) | 38.04 (19.24) |
| 71 | 25 | 0.843 | 0.724 | 19.71 (4.64) | 45.02 (14.62) | 33.83 (9.68) |
| 72 | 12 | 1.771 | 0.724 | 26.73 (6.11) | 72.32 (36.44) | 44.34 (25.4) |
| 75 | 44 | 1.954 | 0.872 | 26.15 (4.38) | 92.85 (35.21) | 22.94 (15.11) |
| 76 | 12 | 0.813 | 0.742 | 23.79 (3.38) | 47.6 (21.16) | 27.93 (18.98) |
| 79 | 21 | 1.875 | 0.926 | 23.57 (4.54) | 101.74 (40.8) | 42.73 (9.74) |
| 80 | 33 | 1.667 | 0.867 | 23.86 (5.2) | 70.68 (18.71) | 43.3 (15.9) |
| 81 | 27 | 0.987 | 0.831 | 24.5 (4.4) | 72.49 (24.56) | 32.21 (24.05) |
| 83 | 5 | 1.120 | 0.907 | 21.84 (4.3) | 75.95 (10.04) | 50.14 (8.26) |
| 84 | 14 | 1.565 | 0.747 | 21.97 (4.07) | 43.66 (15.23) | 37.2 (21.11) |
| 85 | 18 | 1.708 | 0.846 | 22.34 (3.67) | 66.12 (19.08) | 33.51 (20.81) |
| 86 | 4 | 1.031 | 0.648 | 20.37 (3.04) | 42.5 (9.29) | 40.77 (3.39) |
| 87 | 11 | 0.846 | 0.838 | 21.59 (2.78) | 50.26 (12.38) | 44.59 (20.93) |
| 88 | 26 | 2.024 | 0.934 | 23.99 (4.03) | 102.92 (24.9) | 37.06 (13.12) |

**References**

[1] N I Fisher, *Statistical Analysis of Circular Data*, Cambridge University Press, Cambridge, Great Britain, 1993.

[2] J H Zar, *Biostatistical Analysis, 3rd Edition*, Prentice Hall, Upper Saddle River, NJ, 1996.

[3] B L Partridge, The structure and function of fish schools, *Scientific American*, **246**:114—123, 1982.

[4] M Ballerini, N Cabibbo, R Chandelier, A Cavagna, E Cisbani, I Giardina, V Lecomte, A Orlandi, G Parisi, A Procaccini, M Viale and V Zdravkovic, Interaction ruling animal collective behaviour depends on topological rather than metric distance: evidence from a field study, *Proceedings of National Academy of Sciences of the USA*, **105**:1232—1237, 2008.

[5] R Lukeman, Y-X Li and L Edelstein-Keshet, Inferring individual rules from collective behavior, *Proceedings of the National Academy of Sciences of the USA*, **107**:12576—12580, 2010.

[6] Y Katz, K Tunstrøm, C C Ioannou, C Huepe and I D Couzin, Inferring the structure and dynamics of interactions in schooling fish, *Proceedings of the National Academy of Sciences of the USA*, **108**:18720—18725, 2011.

[7] C M Raven, R Shine, M Greenless, T M Schaerf and A J W Ward, The role of biotic and abiotic cues in stimulating aggregation by larval cane toads (*Rhinella marina*), *Ethology* DOI: 10.1111/eth.12645

[8] T M Schaerf, P W Dillingham and A J W Ward, The effects of external cues on individual and collective behaviour of shoaling fish, *Science Advances* 3, 6 e1603201 DOI: 10.1126/sciadv.1603201

[9] T M Schaerf, J E Herbert-Read, M R Myerscough, D J T Sumpter and A J W Ward, Identifying differences in the rules of interaction between individuals in moving animal groups, arXiv preprint arXiv:1601.08202
